# From the Vierteljahrsschrift

**Published:** 1878-05

**Authors:** 


From the Vierteljahrsschrift.—The hitherto announced book, "Filling Teeth and their Extraction," prepared after Taft by Hollander, has made its appearance. So far as a fugitive glance would indicate, a new and valuable work has been placed within the circle of dental practitioners. An extended review will be given at another time.

